# iCodon customizes gene expression based on the codon composition

**DOI:** 10.1038/s41598-022-15526-7

**Published:** 2022-07-15

**Authors:** Michay Diez, Santiago Gerardo Medina-Muñoz, Luciana Andrea Castellano, Gabriel da Silva Pescador, Qiushuang Wu, Ariel Alejandro Bazzini

**Affiliations:** 1 Stowers Institute for Medical Research, 1000 E 50th St, Kansas City, MO 64110 USA; 2 Department of Molecular and Integrative Physiology, University of Kansas Medical Center, 3901 Rainbow Blvd, Kansas City, KS 66160 USA; 3 Present Address: National Laboratory of Genomics for Biodiversity (LANGEBIO), Unit of Advanced Genomics, 36824 Irapuato, Mexico

**Keywords:** Biological techniques, Molecular biology, Translation

## Abstract

Messenger RNA (mRNA) stability substantially impacts steady-state gene expression levels in a cell. mRNA stability is strongly affected by codon composition in a translation-dependent manner across species, through a mechanism termed codon optimality. We have developed iCodon (www.iCodon.org), an algorithm for customizing mRNA expression through the introduction of synonymous codon substitutions into the coding sequence. iCodon is optimized for four vertebrate transcriptomes: mouse, human, frog, and fish. Users can predict the mRNA stability of any coding sequence based on its codon composition and subsequently generate more stable (optimized) or unstable (deoptimized) variants encoding for the same protein. Further, we show that codon optimality predictions correlate with both mRNA stability using a massive reporter library and expression levels using fluorescent reporters and analysis of endogenous gene expression in zebrafish embryos and/or human cells. Therefore, iCodon will benefit basic biological research, as well as a wide range of applications for biotechnology and biomedicine.

## Introduction

The genetic code is degenerate, as most amino acids are encoded by multiple codons^1^. The codons encoding for the same amino acid are called synonymous or silent codons. Long regarded as interchangeable, these codons are not equivalent from a regulatory point of view^1^. Synonymous codons are used with different frequencies in the coding genome, a phenomenon known as codon usage bias^2^. Moreover, synonymous codon substitutions can dramatically affect messenger RNA (mRNA) stability and therefore protein production^1,3–5^. Recent studies have revealed that translation strongly affects mRNA stability in *cis* in a codon-dependent manner in vertebrates^3,6–10^ as well as in other species^4,5,11–15^, a process referred as codon optimality^12^. Codon optimality is the most pervasive mechanism underlying mRNA stability in yeast^16^ and vertebrates^17^. Specifically, to determine the regulatory strength of codon optimality in vertebrates, we have recently developed a machine learning model that predicts mRNA stability based on codon composition^17^. Trained with multiple profiles of mRNA stability for thousands of genes obtained from human^3^ and mouse cells^18^, as well as *Xenopus* and zebrafish embryos^6^, this model has revealed that codon composition is a major determinant of mRNA stability during early embryogenesis and dictates mRNA levels in conjunction with other *cis*-regulatory elements (e.g., microRNA and m^6^A) in human and mouse cells as well as in zebrafish and *Xenopus* embryos^17^. Therefore, we hypothesized that the model could be used as a tool for the design of synonymous coding sequences with differing stability characteristics depending on the desired application.

Existing methods to perform codon optimization are mainly based on codon usage bias^19–22^. Yet, in vertebrates, only weak positive correlations have been observed between codon usage bias and codon optimality^3,6^. For example, the codon usage bias for some amino acids (e.g., Arginine and Threonine) differs drastically from codon optimality^3^. Therefore, a method for codon optimization using codon optimality represents a novel approach for in silico gene design.

Here, we developed a tool named iCodon (www.iCodon.org) that optimizes coding regions with synonymous codon substitutions to increase mRNA stability and therefore protein expression (e.g., to design highly expressed reporters), or deoptimizes sequences with synonymous codon substitutions to decrease mRNA stability (e.g., to design a sequence with decreased expression). iCodon uses a predictive model of mRNA stability^17^ as a guide for supervising the design of sequences. Therefore, iCodon can also be used to visualize the predicted mRNA stability based on the codon composition of any coding sequence. We validated the predictions with a reporter library composed of thousands of sequences in zebrafish embryos, and with particular sequences in both human cells and zebrafish embryos.

In summary, iCodon chooses ideal codons to incorporate into designed coding sequences to reach desired gene expression levels. iCodon is available as an R package (https://github.com/santiago1234/iCodon) and an interactive web interface www.iCodon.org (https://bazzinilab.shinyapps.io/icodon/).

## Results

### iCodon predicts gene expression based on codon composition and designs new variants based on synonymous substitutions

Our machine learning model to predict mRNA stability as a function of codon composition^17^ was trained with mRNA stability profiles from zebrafish and *Xenopus* embryos^6,17^, human cell lines^3^, and mouse embryonic stem cells^18^. Therefore, our model predicts mRNA stability using an arbitrary unit of decay rate, where positive values correlate with stable sequences^17^. We hypothesized that our model could be used to supervise the design of coding sequences with customized host mRNA stabilities based on synonymous codon choice.

**Figure 1:**
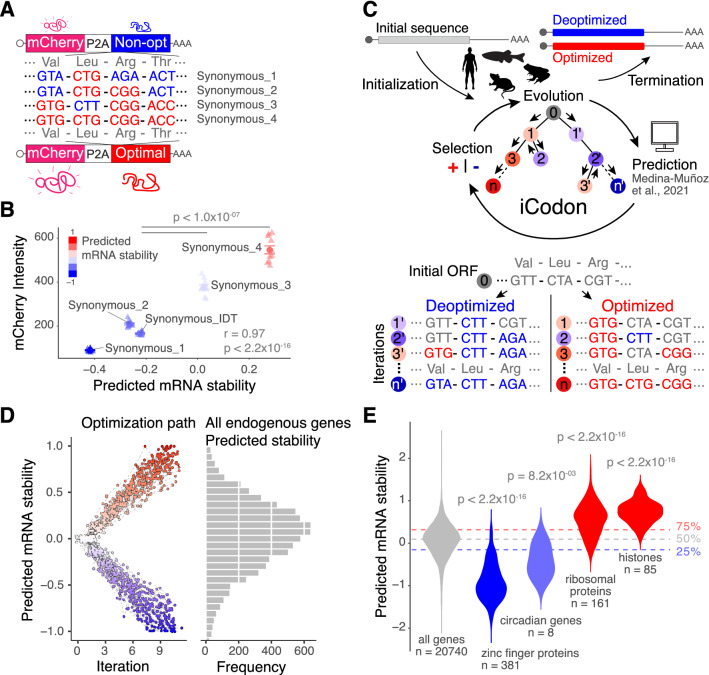
iCodon predicts gene expression based on the codon composition and designs new variants based on synonymous mutations. (**A**) Diagram of the synonymous reporters, differing only in synonymous mutations with different regulatory effects on mRNA stability. Each mRNA contains the coding sequence of mCherry fluorescent protein followed by a ribosome-skipping sequence (P2A) and a coding region that differs in the proportion of optimal and non-optimal codons but encodes the same peptide (synonymous mutations). (**B**) Scatter plot showing that fluorescence intensity of synonymous reporters in 293T transfected cells correlates with predicted mRNA stability (r = 0.97, *p* < 2.2 × 10^−16^, Pearson correlation test). n = 10 for all tested variants. (**C**) Diagram depicting the algorithm for codon optimization, iCodon: An initial coding sequence is provided by the user. Evolution: the algorithm generates variant sequences by introducing random synonymous mutations. Prediction: the machine learning model predicts the mRNA stability of each variant based on the codon composition. Selection: the algorithm selects the sequences with the highest or lowest mRNA stability depending on the direction of optimization. Iteration: this process is repeated multiple times producing an optimization path that generates a gradient in mRNA stability level. Illustrations by Mark Miller. (**D**) A random group of 50 human genes with predicted intermediate mRNA stability was selected and optimized and deoptimized by iCodon. The x-axis is the iteration number, and the y-axis is the predicted mRNA stability. The circles connected by a dash-line show the optimization or deoptimization path for each gene. The histogram on the right is the mRNA stability distribution for endogenous human genes. (**E**) Violin plot showing predictions of mRNA stability of selected groups of genes compared to all genes in the human transcriptome. The horizontal lines show the lower, middle and upper quartiles of the predicted mRNA stability of all genes. *p* values and number of genes (n) are indicated.

First, to test the sensitivity of the predictive model to capture synonymous substitution effects on gene expression, we analyzed previously published reporter sequences designed to generate identical peptides but differing in codon choice^3^. These reporters encode for mCherry followed by a ribosome skipping sequence (P2A)^23,24^ and a variable region enriched in different proportions of optimal (stabilizing) and non-optimal (destabilizing) synonymous codons (Fig. 1A). Importantly, and due to the P2A sequence, mCherry production is independent of potential protein folding differences that may arise for the peptide encoded by the variable region. Previously, we have shown that mRNA levels and fluorescence intensities in transfected human 293T cells correlate with the proportion of optimal codons in the reporter sequences^3^. Here, we found that the model correctly estimated the expression profile of these reporters in transfected human 293T cells (*p* value < 2.2 × 10^–16^, Pearson correlation test) (Fig. 1B). Interestingly, a reporter optimized by IDT’s codon optimization tool with rebalanced codon usage, displayed reduced fluorescence intensity and predicted stability when compared to reporters enriched in optimal codons (Fig. 1B) (Synonymous_3 or Synonymous _4 vs Synonymous_IDT, *p* < 1.0 × 10^–07^, paired t-test). Therefore, our model is able to predict the impact of synonymous codon changes on gene expression.

Next, we created iCodon by coupling the predictive model^17^ to an evolutionary algorithm (Fig. 1C), whereby a given sequence accumulates synonymous mutations through multiple selective iterations (see “Methods” section). The predictive model is used to supervise the selection of new variants with increased (optimized) or decreased (deoptimized) gene expression (Fig. 1C).

We ran a simulation to optimize and deoptimize a group of 50 randomly chosen human genes with intermediate mRNA stability (Fig. 1D). This simulation revealed three important aspects. First, these genes can be optimized or de-optimized to reach the predicted mRNA stabilities of the most or least stable genes in the transcriptome, respectively (Fig. 1D). Second, the algorithm generates a range of intermediate sequences that fall along a gradient of predicted mRNA stabilities (Fig. 1D). And third, the degree of optimization achieved for most genes is predicted to affect mRNA abundance by orders of magnitude ranging from 10 to 100-fold (Supplemental Fig. 1A and B)^25^. These results highlight the potential of iCodon to design coding sequences that display wide stability profiles through synonymous codon substitutions.

### iCodon as an mRNA stability predictor tool

Knowing the predicted stability of particular genes based on the coding sequence can be an entry point to understand its function or regulation mode. Pertinent to this, we have generated transcriptome wide predictions of mRNA stability for all endogenous genes in human, zebrafish, *Xenopus*, and mouse (Supplemental Tables 1, 2, 3, 4). Gene Ontology term analysis of the top 100 most optimal human genes compared to the rest of the transcriptome, revealed an enrichment of pathways related to translation, while analysis of the top 100 most non-optimal genes revealed an enrichment of transcription factors (Supplemental Table 5). Moreover, transcription factors belonging to zinc finger proteins (n = 381) as well as core circadian genes (n = 8) ^26^ displayed a significant lower stability score compared to the stability of the transcriptome (*p* < 2 × 10^–16^ and *p* < 8.2 × 10^–03^, respectively, unpaired t-test) (Fig. 1E). In contrast, histones (n = 85) and ribosomal proteins (n = 161) showed a significant higher stability score compared to the stability of the transcriptome (*p* < 2.2 × 10^–16^, unpaired t-test) (Fig. 1E). Therefore, addressing the predicted stability of a given mRNA could be useful to hypothesize about gene function and evolution. For instance, it can be proposed that core circadian genes might have been under evolutionary pressure to be unstable in order to have oscillatory expression. Therefore, using iCodon to predict the stability of genes based on codon composition uncovered substantial differences in mRNA stability for different gene families.

### iCodon predicts the stability of thousands of mRNAs injected into zebrafish embryos

**Figure 2:**
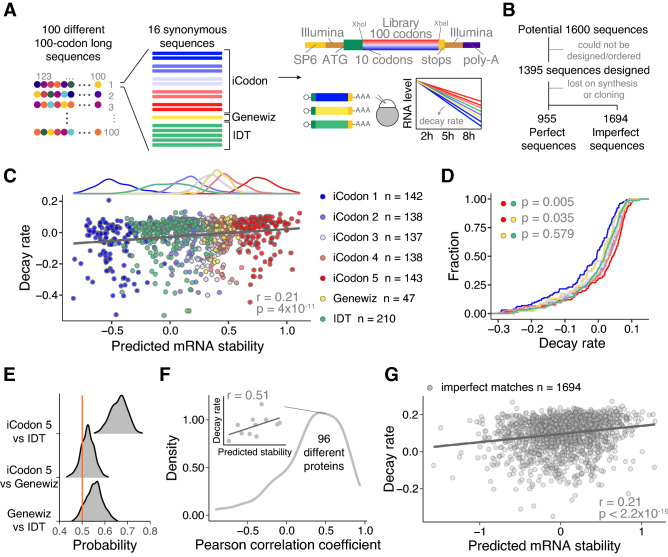
iCodon predicts the stability of thousands of injected mRNAs into zebrafish embryos. (**A**) Schematic of the reporter library. For 100 different 100-codons long proteins, 16 different coding sequences differing only in synonymous codons were designed. For each protein, 10 sequences were designed by iCodon in 5 bins with increasing predicted stability, 5 sequences by the method used by IDT and one for the Genewiz’s method. The sequences were ordered in bulk and cloned into the vector by restriction cloning with XhoI and XbaI, to generate transcripts sharing the same 5′ and 3′UTR containing Illumina adapters (copper) and 27 nt within the translation start site (green). In vitro transcribed mRNAs (SP6 promoter in yellow) were injected into 1-cell stage zebrafish embryos. Reporter mRNA library is analyzed at 2, 5, and 8 h post-injection. (**B**) Pipeline showing the steps to identify the designed reporter sequences (perfect) as well as non-designed sequences (imperfect). (**C**) Top: histogram displaying the frequency of predicted mRNA stability for each group of sequences. Bottom: Scatter plot showing a positive correlation between predicted mRNA stability and decay rate of injected mRNA reporters in zebrafish embryos (n = 955, r = 0.21, *p* = 4 × 10^–11^, Spearman correlation test). The colors indicate the method used to design the coding sequences. (**D**) Cumulative distributions of the decay rate of injected mRNAs reporters into zebrafish embryos designed with different methods (indicated by colors). iCodon 5 versus IDT *p* = 0.005, iCodon 5 versus Genewiz *p* = 0.035, and Genewiz versus IDT *p* = 0.579, unpaired, one-tailed t-test). (**E**) Histogram showing the probability to design the most stable mRNA for the 96 encoded proteins between the indicated methods. Orange line indicates a probability of 0.5. (**F**) Histogram showing the distribution of the Pearson correlation coefficients between the predicted mRNA stability and decay rate of injected mRNA synonymous reporters per protein. Inset shows the correlation between the predicted mRNA stability and decay rate of injected mRNA synonymous reporters for one protein as an example. (**G**) Scatter plot showing a positive correlation between predicted mRNA stability and decay rate of injected mRNA reporters in zebrafish embryos that did not match perfectly with the designed sequences but were observed in at least 2 replicates of each time point and encode for a protein longer than 70 codons (n = 1694, r = 0.21, *p* < 2.2 × 10^–16^, Spearman correlation test).

To test the strength of iCodon to predict and customize mRNA stability in zebrafish embryos, we conducted a massive reporter experiment. First, we designed a library of 1600 sequences containing 100 different 100 amino acid long coding sequences capturing the average composition of the zebrafish proteome, with 10 variants each designed using iCodon to have 5 bins of increasing predicted stability with 2 sequences in each bin (Fig. 2A). The groups were referred as iCodon 1, iCodon 2, iCodon 3, iCodon 4, and iCodon 5, from less to more stable mRNA predictions (Fig. 2A). Additionally, 5 synonymous sequences were designed using 5 independent iterations of IDT’s codon optimization tool and 1 synonymous sequence using Genewiz’s codon optimization tool as this method returned the exact same sequences in each independent run (Fig. 2A). These sequences were cloned downstream of 11 fixed codons to control for similar translation initiation and the reporters contain constant 5’ and 3’ UTR sequences. 1395 of the 1600 designed sequences were ordered, in vitro synthetized mRNA was injected into 1-cell stage zebrafish embryos, and the mRNA decay after 2, 5, and 8 h post injection (hpi) was calculated by targeted RNA-sequencing. 955 reporters (perfect sequences) were detected in all time points (Fig. 2A, Supplemental Fig. 2A, Table 6) and, as expected, all three replicates for each time point presented a strong Pearson correlation (Supplemental Fig. 2B). Next, we calculated the decay rate of all the variants (iCodon = 698, IDT = 210, Genewiz = 47) encoding for 96 different proteins using a first-order reaction model^17^. The predicted mRNA stability estimated by iCodon significantly correlated with the calculated decay rate (r = 0.21, *p* value = 4 × 10^–11^, Spearman correlation test) (Fig. 2C). To compare with other metrics, we used the codon adaptation index, a metric of synonymous codon usage bias used to predict gene expression^27^, but it did not show a significant correlation with the measured decay (r = 0.059, *p* value = 0.067, Spearman correlation test) (Supplemental Fig. 2C). This result illustrates that the iCodon is a good predictor of mRNA stability during zebrafish embryogenesis.

Comparing the three optimization methods, the most stable sequences were generated by iCodon (iCodon 5 vs IDT *p* value = 0.005, iCodon 5 vs Genewiz *p* value = 0.035, unpaired, t-test) (Fig. 2D). As the number of isoforms designed by each of the methods are not the same, we calculated the probability of each method to generate the most stable sequences for the 96 different proteins. Strikingly, iCodon is the tool that most likely produces the most stable sequence compared to Genewiz or IDT (Fig. 2E). Moreover, given that the library encodes for 96 different proteins, for each protein we calculated the Pearson correlation coefficient between the predicted stability and the observed decay of all synonymous sequences (Fig. 2F, individual example inset). As expected, we observed a positive correlation for most proteins (median = 0.39) (Fig. 2F). Therefore, iCodon can be used to generate synonymous coding sequences with a high degree of stability for many different protein sequences.

Intriguingly, we identified expression of 1694 non-perfect matches (i.e., reporter coding sequences with mutations, deletion or insertions) encoding for proteins longer than 70 codons in all time points (Fig. 2B, Supplemental Fig. 2A, Supplemental Table 7). In agreement with the perfect sequences, the predicted mRNA stability of the non-perfect sequences correlated with the decay rate (r = 0.21, *p* value < 2.2 × 10^–16^, Spearman correlation test) (Fig. 2G). Altogether, the analysis of the stability of nearly 3000 mRNAs in a transcription-independent manner (injected mRNA) (Fig. 2C and G), demonstrates that iCodon can be used to design mRNAs with different stability based on the codon composition.

### iCodon generates fluorescent variants with desired protein expression levels

**Figure 3:**
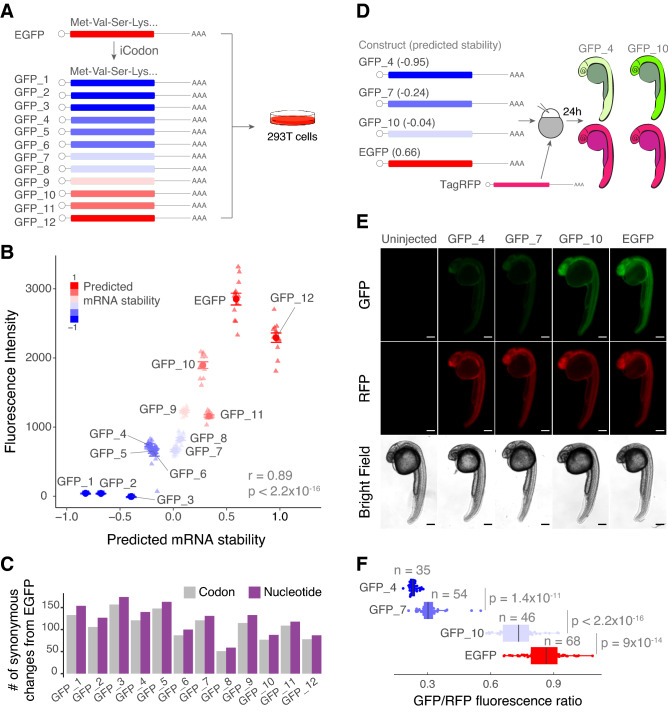
iCodon generates fluorescent variants with desired expression levels. (**A**) Illustration of 12 GFP variants generated by iCodon differing only in synonymous mutations and its predicted mRNA stability. All GFP variants were transfected in 293T cells and the fluorescence was measured by cytometry analysis. (**B**) Scatter plot showing a positive correlation between predicted mRNA stability and GFP fluorescence intensity in 293T transfected cells (r = 0.89, *p* < 2.2 × 10^–16^, Pearson correlation test). n = 12 for all tested variants. (**C**) Barplot displaying the number of codon (gray) or nucleotide (purple) changes in all 12 GFP variants compared to EGFP. (**D**) Four GFP variants were co-injected with TagRFP into 1-cell stage zebrafish embryos and imaged 24 h post injection (hpi). The mRNA stability predictions by iCodon in zebrafish are indicated in brackets. iCodon predictions are slightly different for zebrafish compared to humans as codon optimality is not identical between these two species. (**E**) Microscopy images of injected zebrafish embryos after 24 hpi. Scale bars represent 200 µm. (**F**) Quantification of the differences of fluorescence intensity of GFP relativized by RFP fluorescence from injected zebrafish embryos. *p* values and replicates (n) are indicated.

After analyzing the stability of > 2500 mRNAs injected into zebrafish embryos (Fig. 2), we tested the potential of iCodon to produce sequences with different protein expression levels. Using iCodon, we optimized and deoptimized EGFP (enhanced Green Fluorescent Protein) and generated twelve GFP variants ranging in different levels of codon optimality for human cells (Fig. 3A). We found that the predicted mRNA stability correlated with protein fluorescence intensities observed in transfected 293T human cells (r = 0.89, *p* value < 2.2 × 10^–16^, Pearson correlation test) (Fig. 3B). Nearly 50-fold differences in intensity were observed between the GFP variants designed by iCodon with the highest and lowest expression levels (Fig. 3B). The fluorescence intensity for the most optimal GFP variant (i.e., GFP_12) was not as strong as that observed for EGFP (Fig. 3B). However, this result was not surprising, as EGFP has been extensively optimized for increased fluorescence^28,29^. Yet, this GFP variant (GFP_12) differs from EGFP by 87 nucleotides and 78 codons (Fig. 3C and Supplemental Fig. 3A), without affecting fluorescence intensity drastically (EGFP vs GFP_12 fold-change = 1.24, *p* value = 2.0 × 10^–07^, paired t-test). Moreover, from EGFP, 131 nucleotides (121 codons) were changed to create one of our neutral GFPs (GFP_7), and 127 nucleotides (106 codons) mutations generated our dimmest, destabilized GFP (GFP_2) (Fig. 3C and Supplemental Fig. 3A). Given that EGFP is already codon optimized, more changes were required on average to create the destabilized sequences and these results further show that the type of substitution is more important than the number of mutations to affect gene expression.

Some GFP variants, such as GFP_3 and GFP_11, did not necessarily follow the expected expression level (Fig. 3B). For example, GFP_3 did not produce any detectable GFP expression, which illustrates that there are likely other features (e.g., nucleotide sequence, RNA structure, etc.) affecting protein expression, highlighting the nonequivalent nature of synonymous substitutions.

Next, to test whether the iCodon predictions correlated with fluorescence expression levels in an in vivo model (zebrafish embryos), variants that displayed low (GFP_4), medium (GFP_7) and high (GFP_10) expression in transfected human cells (Fig. 3A,B) were selected. mRNA of each variant, including EGFP, was co-injected with TagRFP mRNA as an internal control into 1-cell stage zebrafish embryos and the GFP/RFP ratio was measured at 24 h post injection (hpi) (Fig. 3D). The GFP variants displayed profiles of fluorescence intensity (Fig. 3E,F) similar to those observed in human cells (Fig. 3B), which correlated with their predicted stability (r = 0.86, *p* value < 2.2 × 10^–16^, Pearson correlation test). Together, these results illustrate the ability of iCodon-specified synonymous substitutions to modulate protein expression in both human cells and zebrafish embryos.

### iCodon improves performance of fluorescent AausFP1 variants for expression in vertebrates

**Figure 4:**
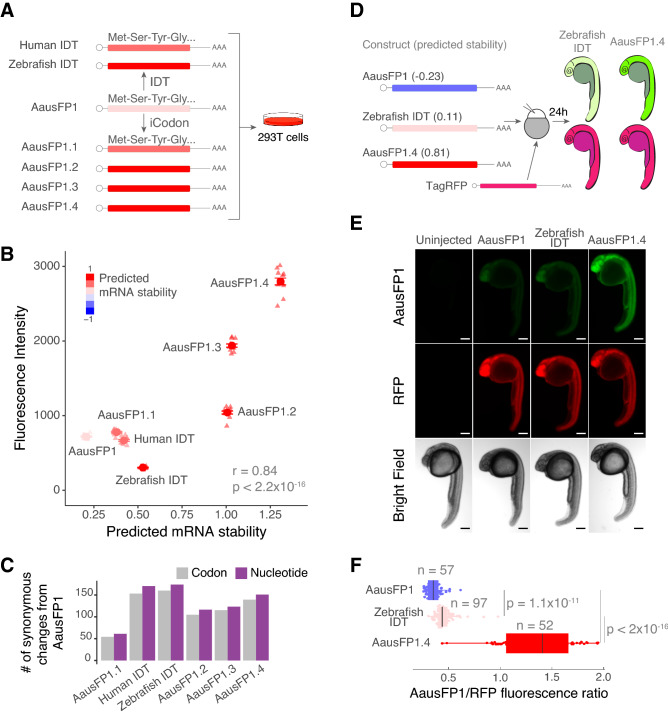
iCodon optimizes fluorescent AausFP1 variants for expression in vertebrates. (**A**) Diagram of the AausFP1 variants optimized by iCodon or by IDT Codon Optimization Tool that were transfected in 293T cells. (**B**) Scatter plot showing a positive correlation between predicted mRNA stability and AausFP1 fluorescence intensity in 293T transfected cells (r = 0.84, *p* < 2.2 × 10^–16^, Pearson correlation test). n = 12 for all tested variants. (**C**) Bar plot showing the number of codon (gray) or nucleotide (purple) changes in AausFP1 variants compared to the original sequence. (**D**) Three AausFP1 variants were co-injected with TagRFP into 1-cell stage zebrafish embryos and imaged 24 h post injection (hpi). The mRNA stability predictions by iCodon in zebrafish are indicated in brackets. (**E**) Microscopy images of injected zebrafish embryos after 24 hpi. Scale bars represent 200 µm. (**F**) Quantification of the differences of fluorescence intensity of AausFP1 relativized by RFP fluorescence from injected zebrafish embryos. *p* values and replicates (n) are indicated.

We next tested whether iCodon can improve the performance of AausFP1, a recently identified fluorescent protein from *Aequorea. cf. australis*^30^. This protein is reported to be 5-fold brighter and more photostable than EGFP^30^ but has not yet been optimized for vertebrate expression. Starting with the reported AausFP1 coding sequence^30^, four optimized versions were designed by iCodon, as well as two variants designed by the rebalancing of codon usage (IDT Codon Optimization Tool, www.idtdna.com) for zebrafish and human expression (Fig. 4A). Similar to the GFP results (Fig. 3), the stability predicted by iCodon correlated with AausFP1 fluorescence intensities observed in transfected 293T human cells (r = 0.84, *p* value < 2.2 × 10^–16^, Pearson correlation test) (Fig. 4B). Strikingly, a nearly 4-fold change in fluorescence intensity was observed between the original AausFP1 coding sequence and a AausFP1.4 variant optimized by iCodon in 293T cells (*p* value = 1.6 × 10^–14^, paired t-test) (Fig. 4B). In contrast, small differences in fluorescence intensity were observed between the original AausFP1 and the variant optimized by IDT (AausFP1vs Human IDT, *p* value = 0.01, paired t-test) in 293T cells (Fig. 4B). A variant optimized for zebrafish actually displayed reduced fluorescence intensity in transfected human cells (AausFP1vs Zebrafish IDT, *p* value = 1.1 × 10^–11^, unpaired t-test) (Fig. 4B). Similar to the EGFP experiment (Fig. 3), the types of codon substitutions, rather than overall number of substitutions, had the greatest effect on gene expression. For example, the brightest variant optimized by iCodon (AausFP1.4) contained less substitutions than a weaker (lower fluorescence intensity) variant with rebalanced codon usage (Fig. 4C and Supplemental Fig. 3B).

Next, we tested whether the iCodon predictions of AausFP1 variants for zebrafish correlate with fluorescence expression levels in zebrafish embryos. Three AausFP1 variants (mRNA) were co-injected with TagRFP mRNA as internal control into 1-cell stage zebrafish embryos and AausFP1/RFP ratio was measured at 24 hpi (Fig. 4D). We observed a positive correlation between the stability predicted by iCodon and the AausFP1 fluorescence intensities observed in injected zebrafish embryos (r = 0.86, *p* value < 2.2 × 10^–16^, Pearson correlation test) (Fig. 4E,F). Moreover, the optimized variant displayed nearly 4-fold more fluorescence (AausFP1/RFP ratio) than the original AausFP1 (AausFP1.4 vs AausFP1, *p* value < 2.2 × 10^–16^, unpaired t-test) and close to 3-fold more fluorescence when compared to the variant optimized by IDT (AausFP1.4 vs Zebrafish IDT, *p* value < 2.2 × 10^–16^, unpaired t-test) (Fig. 4E,F). In sum, iCodon is able to improve the expression of heterologous coding sequences for use in vertebrate systems.

### Optimized endogenous variant rescues loss-of function phenotypes

**Figure 5:**
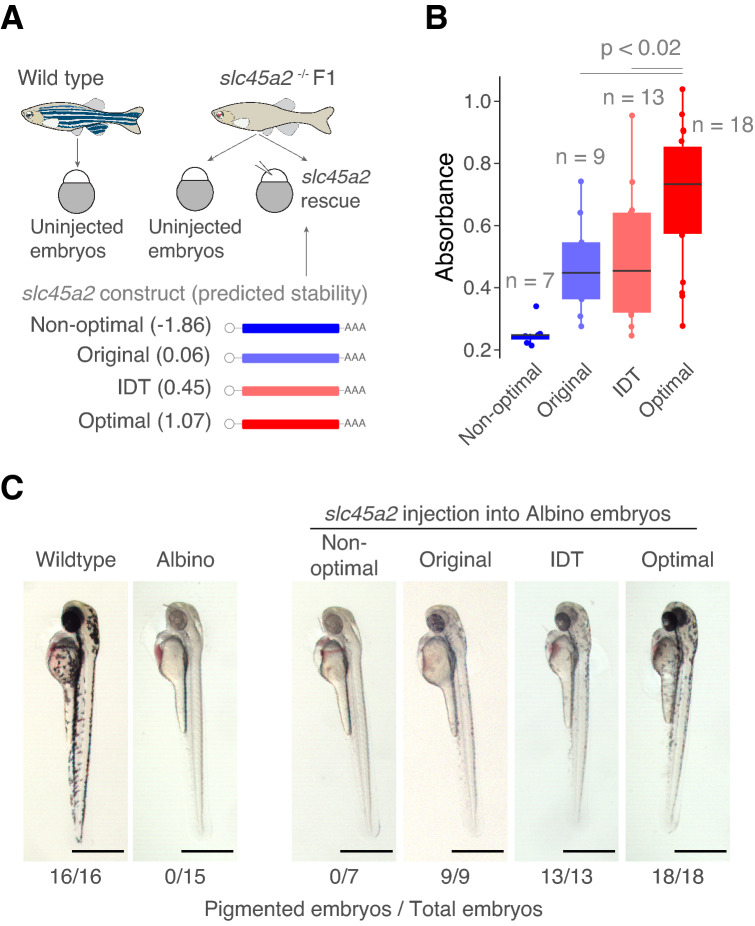
Optimized endogenous variant rescues loss-of-function phenotypes. (**A**) Schematic representation of the rescue experiment in zebrafish embryos. Variants of the *slc452* gene (melanin pigmentation) were injected into loss-of-function *slc45a2* knockout zebrafish embryos (albino phenotype, lack of pigmentation). The predicted mRNA stability of the variants is indicated. Illustrations by Mark Miller. (**B**) Quantification of the absorbance of the pigmentation as a measure of the amount of phenotype rescue. *p* values and replicates (n) are indicated. (**C**) Microscopy images of zebrafish embryos showing a degree of loss-of-pigmentation phenotype rescue by codon optimized variants compared to the albino fish. Wildtype embryo is shown in the left. Scale bars represent 700 µm. The numbers reflect the proportion of embryos that showed melanin pigmentation.

We next hypothesized that iCodon could enhance the expression of coding sequences from within the host genome. Specifically, we tested whether iCodon optimization could improve the ability of endogenous coding sequences to rescue loss-of-function phenotypes that might depend on protein dosage. For this, we first generated a zebrafish line lacking melanin pigmentation (albino phenotype) by targeting gene *slc45a2* with CRISPR/Cas9^31^. Next, we designed four *slc45a2* mRNA variants to inject into zebrafish embryos: the original *slc45a2* coding sequence (original), as well as one variant optimized by IDT (IDT) and two variants predicted by iCodon to have high and low stability (optimal and non-optimal) for zebrafish expression (Fig. 5A). In vitro transcribed mRNAs for each *slc45a2* variant were injected into 1-cell stage albino zebrafish embryos. Pigmentation was minimally rescued by the original *slc45a2* variant and not rescued by the non-optimal variant after 48 hpi. In contrast, embryos injected with either optimized variant (IDT or iCodon) displayed pigmentation after 48 hpi. Quantification of the pigment absorbance revealed that the variant optimized by iCodon displayed the highest level of rescue (*p* < 0.02, unpaired t-test) (Fig. 5B,C). These results highlight the ability of iCodon optimization to improve the ability of endogenous coding sequences to rescue loss-of-function mutants.

### Steps to use iCodon

**Figure 6:**
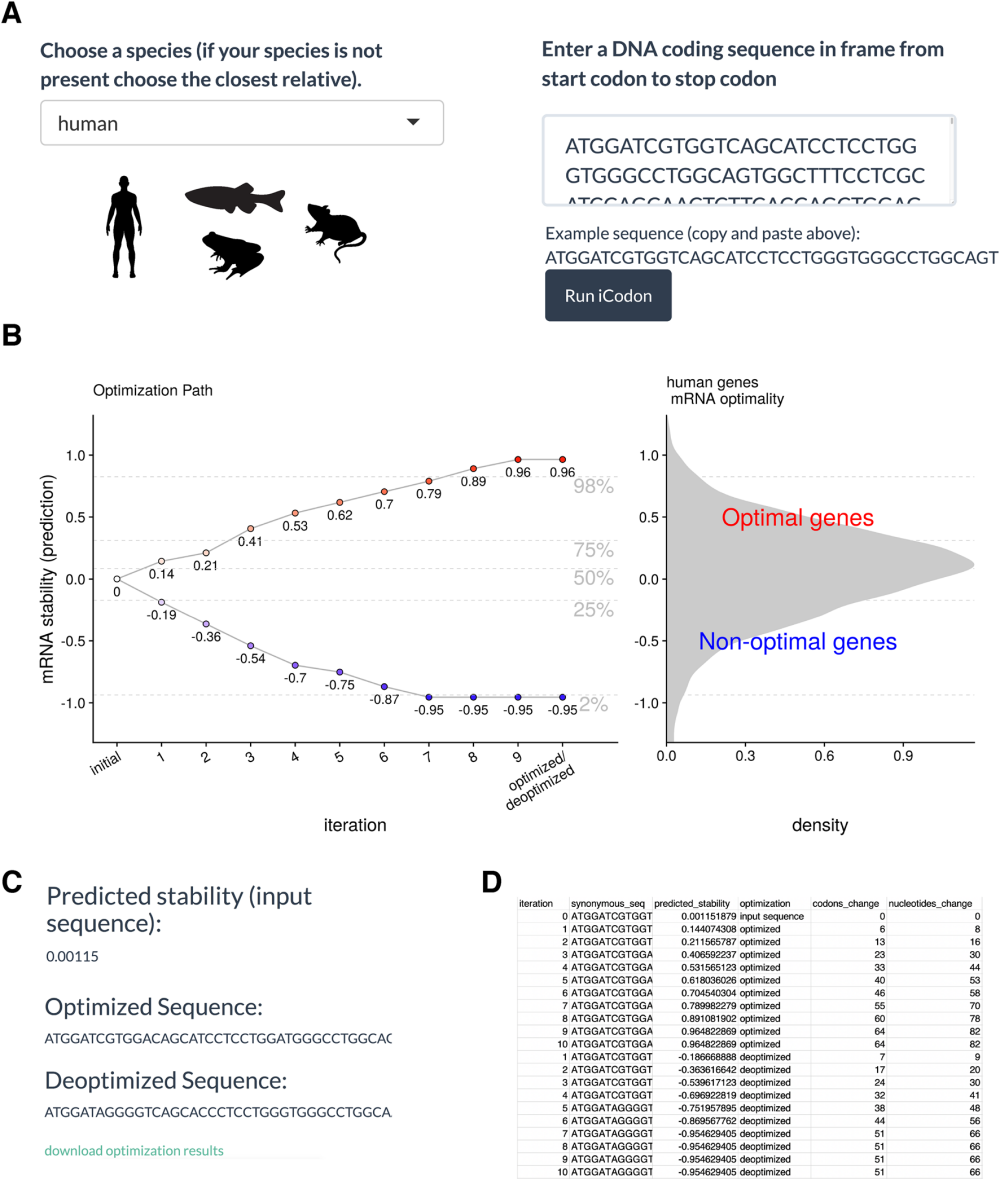
iCodon user steps. (**A**) The user needs to select between four species (human, mouse, zebrafish or *Xenopus*). The coding sequence (A, T, G and C) needs to be pasted into the box indicated and the Run iCodon box needs to be clicked. Illustrations by Mark Miller. (**B**) The scatter plot will show the original sequence in grey with its predicted stability. Each of the optimized (red) or deoptimized (blue) sequences with each respective stability score will be displayed. A histogram of the mRNA stability distribution of endogenous genes of the selected species is shown to use as a reference for the designed variants. (**C**) The original sequence, as well as all designed variant sequences, stability scores and nucleotide/codon changes with respect to the original sequence will be provided in a file by clinking “download optimization results”. (**D**) Example table of the downloaded iCodon results.

The iCodon interactive web interface is available at www.icodon.org as well as at https://bazzinilab.shinyapps.io/icodon/. The user can provide a coding sequence (A, T, C and G, case-insensitive) and select the relevant species (human, mouse, zebrafish or *Xenopus*) (Fig. 6A). A warning message is displayed if the pasted sequence is not a multiple of three, contains an internal stop codon, or does not contain a terminal stop codon. The results are displayed graphically, showing the degree of optimization achieved (Fig. 6B), and the original sequence is plotted as a white dot with the predicted stability. Nine optimized variants are shown in red and nine deoptimized sequences in blue, with their respective predicted stabilities indicated. Finally, by selecting the “download optimization results” link (Fig. 6C), the user can retrieve the optimized and deoptimized coding sequences, as well as their predicted stabilities, number of codons and nucleotides changes in text format ready for downstream synthesis applications, (csv extension) (Fig. 6D). Moreover, if the user wishes to study multiple sequences, iCodon can be run directly in R https://github.com/santiago1234/iCodon (Supplemental file 1).

## Discussion

The protein production outcome can be collectively influenced by regulatory elements in the promoter, 5′UTR, coding sequence, and 3′UTR^32^. Further, codon composition affects transcription, mRNA structure, mRNA stability, translation initiation, elongation rate, and protein folding (reviewed by^33^). Here, we leveraged the impact of codon composition on mRNA stability to show that iCodon can design in silico sequences with increased or decreased stability profiles by codon synonymous substitutions for vertebrates (Figs. 2, 3, 4 and 5).

**Figure 7:**
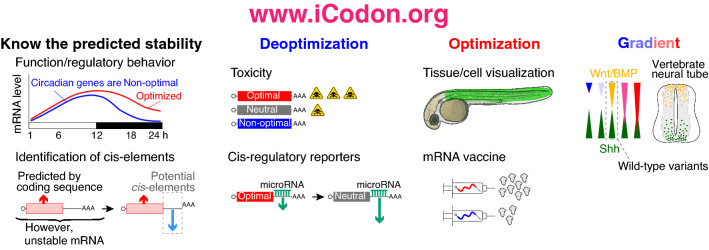
iCodon potential applications. iCodon can be used to uncover gene expression patters from the coding sequence; or to identify cis-regulatory elements. iCodon can be used to design sequences with neutral mRNA stability, these sequences will be more responsive to other regulatory elements (i.e., miR-430). Exogenous genes that are wanted to be expressed in a vertebrate model organism could result toxic for the cell; by designing coding sequences with a decreased expression, the toxicity level can be reduced. Tissue/cell visualization: fluorescent proteins found in another organism can be optimized for expression in vertebrate species. Injected mRNA (zebrafish embryos) or mRNA vaccine design can be codon optimized to increase the mRNA stability and expression. Expression gradient: iCodon has the potential to design a wide variety of coding sequences, which will show different gene expression patterns.

We anticipate a number of applications for iCodon. First, and at the most basic level, users can interrogate the stability of endogenous genes based on their coding sequence. Knowing the relative stability of a gene of interest can provide potential insight into its biological role and/or evolution. For example, we observed that core circadian clock genes tend to be unstable in the human transcriptome (Figs. 1C and 7), as observed for Neurospora, where non-optimal codon composition is essential for circadian clock function^34^. Additionally, our observation of ribosomal proteins being optimal in human recapitulates what has been observed in yeast^12^. The conserved optimality/non-optimality of these gene sets suggests that there has been evolutionary pressure to maintain the codon optimality of genes involved in fundamental biological processes such as translation or circadian rhythm.

Second, we have recently shown that an accounting of codon-mediated effects on mRNA stability can help to identify other contributing *cis*-regulatory elements^17^. For instance, microRNAs and RNA modifications such as m^6^A target a subset of maternal mRNAs for degradation during embryogenesis^35–40^, whereas other modifications (e.g., m5C) are associated with stabilization of maternal RNAs^41^. Interestingly, we have observed higher enrichment of destabilizing (miR-430 and m^6^A) or stabilizing (m^5^C) *cis*-regulatory elements in the 3′UTRs of genes in which codon optimality did not explain observed stability when compared to genes in which codon composition was highly predictive of observed stability^17^. Therefore, by controlling for the predicted mRNA stability based on codon composition, other gene regulatory networks that influence mRNA stability can be discovered (Fig. 7).

Third, users can re-design the codon composition of reporter mRNAs and transgenes for specific downstream purposes. For example, we have observed that microRNA and m^6^A regulation dictate mRNA stability in conjunction with codon optimality^17^. Specifically, microRNA or m^6^A targets with more optimal coding sequences are more stable than non-optimal target mRNAs^17^. However, in genes highly enriched in optimal or non-optimal codons the microRNAs (miR-430/-427) targeting efficacy is reduced during embryogenesis^17^. Therefore, researchers might want to evaluate the codon optimality of classical GFP, mCherry or luciferase reporters and subsequently deoptimize them to reach an average level of stability^3^ (Fig. 7**)**. Such re-tooling may be well-advised, as some ‘highly-stable’ reporter mRNAs may not reflect the majority of endogenous stability profiles. Additionally, reducing the level of gene expression can be desired to reduce the toxic effect of highly expressed proteins (Fig. 7).

Fourth, users can design transgenes to be more highly expressed for a myriad of applications, including protein visualization (e.g., GFP or AausFP1, Figs. 3 and 4), mRNA knock-down^42^, genome editing^43^ and loss-of-function rescue (Fig. 5). Through iCodon optimization, the amount of mRNA required to rescue loss-of-function phenotypes can be reduced, thereby avoiding toxic effects associated with the introduction of high amounts of exogenous RNA^44^. While screening random synonymous substitution libraries for variants with desired expression levels is a valid approach^45^, the number of potential variants could be unfeasibly large, depending on the coding sequence size. Our results show that in zebrafish embryos and human cells, iCodon outperforms both IDT’s and Genewiz’s codon optimization tool to create the most stable sequences and/or most highly expressed variants (Figs. 2, 3, 4 and 5). In agreement with no significant correlation between the stability of the injected mRNA and codon adaptation index (Supplemental Fig. 2B), a recent work showed that codon optimality is a better predictor of mRNA stability than codon usage in zebrafish^46^. Therefore, iCodon provides a practical first step toward more targeted solutions.

Fifth, users may employ iCodon to design a range of variant transcripts that have slightly different expression profiles in order to, for example, measure dosage effects of morphogens during development (e.g., Sonic hedgehog (Shh), BMP or Wnt) (Fig. 7). Additionally, iCodon can simply be used when genes from one species need to be expressed in a heterologous context (e.g., AausFP1 from *Aequorea. cf. australis* into human cells and zebrafish embryos, Fig. 4).

Finally, we envision that iCodon can contribute to the design of RNA-based therapeutics (e.g., mRNA vaccines) (Fig. 7), in which increased stability and expression may correlate with stronger efficacy (e.g., stronger immune response) and/or smaller doses^47–50^.

Past work has demonstrated that both mRNA stability and translation efficiency are driven by codon optimality in zebrafish^6^, therefore we do not exclude the possibility that iCodon is generating more stable sequences that are also more efficiently translated. And while iCodon predictions correlated with observed gene expression for most genes tested (Figs. 2, 3 and 4), we have observed that particular variants have not necessarily followed the expected expression level. As stated above, there is other regulatory information encrypted in the coding sequence that can affect mRNA stability and gene expression, such as translational ramp^51^, lysine homopolymers^52^, and/or protein folding/activity^53^, just to mention a few. Furthermore, in the future, it would be interesting to study the unexplained variation between expected and observed stability of our library (Fig. 2) to identify other regulatory features encrypted in the coding sequence. Therefore, we recommend designing more than one synonymous sequence, as optimization requires experimental validation.

## Conclusion

In summary, iCodon provides a simple tool for the scientific community to interrogate mRNA stability of their genes of interest based on codon composition, and to design strategies to modulate expression levels in vertebrates through codon optimization or deoptimization.

## Methods

### Evolutionary algorithm for codon optimization

The number of possible synonymous sequences coding for the same peptide is astronomically large. To solve the problem of finding a particular sequence with a target mRNA stability, we developed a genetic algorithm^54^. This algorithm operates on three steps (Fig. 1C):

- *Initialization Step* An initial coding DNA sequence is provided together with a vertebrate species (human, mouse, zebrafish, or *Xenopus*). Also, a fixed threshold ***t*** is set (*t* = 1 by default). This threshold represents the maximum predicted mRNA stability that a sequence can achieve.
- *Evolution Step* *N* daughter sequences are generated (*N* = 10 by default). For each random sequence, a proportion of *p* codon positions (*p* = 0.05 by default) are selected uniformly at random (excluding start and stop codons). For each of these positions, a synonymous codon is randomly selected to introduce a synonymous mutation. We created a custom sampling distribution for selecting random synonymous codons. For the optimization process the distribution samples optimal codons with higher frequency and for the deoptimization process non-optimal codons are sampled with higher frequency. This sampling distribution was generated by ranking the codon stabilization scores^3,6,12^ and then applying the softmax function.
- *Selection Step* The fitness of each daughter sequence is evaluated, and the fittest sequence is selected based on whether the daughter follows the optimized or deoptimized path. The fitness of the sequence is the predicted mRNA stability based on the codon composition^17^.

This process is iterated ***m*** times (***m*** = 10 by default) producing an optimization path. In each iteration the fittest sequences are kept (i.e., the most optimal and the most non-optimal). The optimization path produces a gradient in mRNA stability level. If the predicted mRNA stability of the fittest daughter sequence is more than the threshold ***t***, the process will stop generating new daughter sequences. The last sequences in the iteration correspond to the most optimized or deoptimized variants. The default parameters were selected by heuristic observations.

iCodon is implemented in R (version ≥ 3.6.2), and the source code is available from GitHub https://github.com/santiago1234/iCodon .

### Gene ontology term analysis

The Gene Ontology term analysis was conducted on the GOrilla website http://cbl-gorilla.cs.technion.ac.il/^55^, with two unranked lists of genes as running mode. Either the Top 100 optimal or non-optimal genes were selected as target, and the rest of the genes were selected as background.

### Transcriptome analysis

The coding genes in each transcriptome were downloaded from biomart (Ensembl Genes 102, human = GRCh38.p13, *Xenopus* = Xenopus_tropicalis_v9.1, mouse = GRCm38.p6, and zebrafish = GRCz11)^56^. The longest isoform for each gene was kept. The predicted mRNA stability for each gene was computed with iCodon^17^ (Supplemental Tables 1, 2, 3, 4).

For the stability comparison between groups of genes in human (Fig. 1E), circadian genes were chosen based on the core components of the circadian clock^26^, histones and zinc finger proteins were obtained from Uniprot under the search terms ‘histone’ and ‘zinc finger protein’, and curated manually, and ribosomal protein genes were obtained from the Gene Ontology term structural constituent of ribosome (GO:0003735).

### Reporter library design and preparation

First, we generated 10 random protein sequences of 100 amino acids by sampling each amino acid according to the average usage in the zebrafish proteome. Next, for each of these, we generated 10 new sequences by generating random permutations of the original sequence (i.e., same amino acids but in a different order). For each of these 100 proteins, we obtained a DNA sequence that was introduced in two independent runs of iCodon to reach five target values of predicted mRNA stability: − 0.99, − 0.16, 0.08, 0.31, and 0.77. This process was repeated for each DNA sequence, obtaining 10 synonymous sequences for each protein, to a total of 1000 sequences designed by iCodon. Then, the amino acid sequences were introduced in Genewiz and IDT codon optimization tools. For each tool, we run the optimization 5 independent times to obtain multiple optimized sequences. However, Genewiz returned the exact same sequences in each independent run. In total, we could design 1395 sequences that were synthesized as oligo pools (IDT). The oligo pools were amplified by PCR (forward oligo: GAGGAGCTGTCCCTCGAG and reverse oligo: CCGATCCTAGCTACCTATCTAG), and both the pCS2-based vector backbone and the PCR were digested with XhoI and XbaI restriction enzymes (NEB), and then ligated to obtain the final construct (Fig. 2A). The library was linearized and then used as a template for an IVT reaction (SP6 mMessage mMachine). The purified IVT was quantified using the Qubit Fluorometric Quantification and resolved in an Agilent RNA 6000 Nano bioanalyzer chip.

### Reporter library injection and sample preparation

10 pg of library mRNA were injected into 1-cell stage zebrafish embryos in 3 independent replicates. Each replicate utilized an independent needle, injection mix, and breeding. 25 embryos were collected at 2, 5, and 8 h post-injection (hpi) in Buffer RLT (QIAGEN RNeasy kit), followed by vortexing for 1 min and stored at − 70 °C until extraction. RNA was thawed at 37 °C, extracted following the manufacturer’s protocol, and eluted in 50 μL. mRNA was isolated with Dynabeads oligo-(dT) mRNA purification kit following the manufacturer’s protocol. mRNA was eluted in 10 μl of 10 mM Tris-HCl, pH 7.5. 5 μL of purified mRNA were used for cDNA synthesis using the SuperScript IV Reverse Transcriptase kit, utilizing a specific oligo that binds to the 3′ Illumina adapter (GTAGTGACTGGAGTTCAGAC). The cDNA was cleaned with the Qiagen MinElute PCR purification kit and eluted in 20 μL of water. We tested different PCR cycles to amplify the library using specific oligos that target the surrounding Illumina adapters. We selected 16 cycles for the samples at 2 and 5 hpi, and 18 cycles for 8 hpi. The barcoded libraries were cleaned using AMPure XP beads and resuspended in 20 μL of buffer EB (Qiagen). The libraries were sequenced in an Illumina MiSeq instrument 250 bp pair-end with 10% of PhiX as spike-in.

### Reporter library analysis

Paired end reads were merged with FLASH^57^ using default parameters, reads that could not be merged were discarded. Next, using Cutadapt^58^, the constant regions (5′ = ATGGTGAGCAAGGGCGAGGAGCTGTCCCTCGAG and 3′ = TCTAGATAG) were removed to keep only the variable region. Reads without these regions were discarded (Supplemental Fig. 2A). Then, reads were separated by length (read length = 297, expected length based on sequence design) and mapped them to the library with salmon to quantify expression at each time point^59^. Mapped reads represent the perfect sequences that we designed (Supplemental Fig. 2A). Then, the reads that were not mapped (read length = 297, nucleotide substitutions) were merged with the reads that have a different sequence length (read length < 297 or > 297, insertions and/or deletions). All these reads were called the “imperfect set”. The constant regions that were removed in the first steps of the analysis were added back and therefore every unique read contains a coding sequence which starts in the first ATG (start codon). The codon composition was counted as consecutive triplet until it finds a stop codon. The occurrences of each unique read were counted at every time point to calculate transcript abundance (transcripts per million) (Supplemental Fig. 2A). The library mRNA decay rate was estimated as described before^17^. In brief, we employed a first-order reaction model that describes the relationship between the mRNA abundance (transcripts per million) and time after injection. From this model, the slope is the mRNA decay rate. For the perfect sequences, only sequences that were identified in all the samples (all replicates and all time points) were computed (i.e., 9 data points are used to estimate mRNA stability). For the imperfect sequences, a minimum threshold of mRNA abundance was set (at least 20 reads in each replicate) and they needed to be detected in at least 2 out of 3 replicates for each time point (i.e., at least 6 data points are used to estimate mRNA stability) (Supplemental Fig. 2A). For all perfect and imperfect sequences, the predicted stability based on the coding sequence was calculated with iCodon.

### Probability plot

To calculate the probability of each optimization method to generate the most stable sequences, we first selected proteins for which variants had been optimized using each tool (i.e., iCodon 5, IDT, and Genewiz). For each protein, we randomly selected a variant designed by each tool and compared their decay rate in a pair-wise fashion. A value of 1 was assigned if iCodon 5 showed higher decay rate than its counterpart, and a value of 0 if the decay was equal or lower than its counterpart. Next, the average of these values was calculated. This process was repeated 300 times, and the distribution of the averages computed in the repetitions of random selection and comparison is shown in Fig. 2E.

### Variants clones

Sequences designed by either iCodon or IDT Codon Optimization Tool were synthetized by IDT and cloned into pCS2 backbones using conventional restriction cloning or HiFi DNA Assembly (NEB). Synonymous reporters (Fig. 1A, B) were previously constructed^3^ with the exception of the IDT reporter that was cloned as described above. All sequences can be accessed in Supplemental Table 6.

### Human cells transfection experiments

293T cells were obtained from the Tissue Culture core facility at the Stowers Institute for Medical Research. For transfection, cells were plated in 96-well plates at a relatively low passage, cultured with DMEM media, 10% FBS, L-glutamine and penicillin/streptomycin, and set overnight to reach 70% confluency the day of transfection. Prior transfection, all plasmids were quantified using the Qubit Fluorometric Quantification. 293T cells were transfected using Lipofectamine 3000 based on the manufacturer's instructions, and 24 h post transfection, cells were collected for cytometry analysis. The fluorescence intensity of the cells was quantified in a ZE5 Cell Analyzer, using lasers and detectors for GFP (488/510) and mCherry (587/610). The cytometry data .fsc file were analyzed with FCS Express 7, and the median intensity of the cells was used to represent fluorescence intensity.

### mRNA in vitro transcription

Plasmids carrying the different constructs employed in this study were first digested to linearize the DNA and then used for in vitro transcription using the mMESSAGE mMACHINE® Kit, following manufacturer’s protocols. Prior injections, mRNA was quantified using the Qubit Fluorometric Quantification.

### Zebrafish Embryo Injection and Image Acquisition

Optimized and deoptimized mRNAs encoding EGFP (100 pg), AausFP1 (100 pg), or *slc45a2* (200 pg) were co-microinjected with TagRFP (100 pg) in zebrafish embryos at 1-cell stage (see figure legends for details in each experiment). Injected embryos were collected, mounted for imaging in low melting point agarose as described in^60^, analyzed and quantified between 24 h and 2 days post injection depending on the experiment.

Zebrafish embryo fluorescent pictures were analyzed using a Nikon TI2-E inverted microscope, photographed with a Photometrics Prime 95B-25MM back-illuminated sCMOS camera using laser for GFP (491/535) and RFP (574/615) and images processed with NIS-Elements Advanced Research Package software. Images were further processed in Fiji. AausFP1, GFP and RFP fluorescence were quantified using Fiji (Image J) software by performing sum slices z-projection, selecting the embryo’s trunk region and measuring mean pixel value in the same manner for all conditions.

Zebrafish embryo pigmentation phenotype pictures were analyzed using a ‘Zeiss Lumar V12 steREO’ microscope with same conditions for all the injected embryos and images processed with MicroManager software version 1.4.23 ^61^. Pigmentation was quantified in Fiji by measuring mean pixel intensity in the eyes and calculating brightfield absorbance with the following formulae: Absorbance = − log(T), where T = (eye intensity − dark blank)/(light blank − dark blank).

### Albino zebrafish line

To generate the albino loss-of-function zebrafish, 1-cell stage embryos were injected with Cas9 mRNA along with gRNA targeting the coding sequence of *slc45a2* as previously reported^31^. The F0 lacked melanin pigmentation and were crossed to obtain the F1 used for the experiments of this study.

### Zebrafish maintenance

Zebrafish experiments were done according to the IACUC approved guidelines. Zebrafish embryos collected for microinjections were coming from random parents (AB, TF and TLF, 6–25 months old) mating from 3 independent strains or from random parents (*slc45a2*^-/-^ in AB, TF and TLF background, 6–25 months old) mating from 1 strain. The embryos were pooled from random 12 males and 12 females for each set of experiments, or random 8 males and 3 females from the *slc45a2*^-/-^ parents.

### Statistical analysis

All statical analyses were conducted in R version 4.0.2. Displayed data results from at least two independent experiments, except for the massive reporter library experiment.

## Supplementary Information

Supplementary Information 1.

Supplementary Information 2.

Supplementary Information 3.

Supplementary Information 4.

Supplementary Information 5.

Supplementary Information 6.

Supplementary Information 7.

Supplementary Information 8.

Supplementary Information 9.

## Data Availability

Original data underlying this manuscript can be accessed from the Stowers Original Data Repository at http://www.stowers.org/research/publications/libpb-1591. Sequencing data have been deposited in the NCBI Gene Expression Omnibus, GSE207584. The source code and datasets generated during the current study are available in the GitHub repository, https://github.com/santiago1234/iCodon. The iCodon interactive web interface is available at www.iCodon.org or https://bazzinilab.shinyapps.io/icodon/.

## Abbreviations

mRNA: Messenger RNA
miR: MicroRNAs
m^6^A: N6-methyladenosine
m^5^C: 5-Methylcytosine
P2A: 2A ribosome skipping sequence
GFP: Green fluorescent protein
EGFP: Enhanced green fluorescent protein
AausFP1: *Aequorea. cf. australis* Fluorescent protein 1
UTR: Untranslated regions
