# Supplementary material for: iCodon customizes gene expression based on the codon composition
Source: Sci Rep. 2022 Jul 15;12:12126. doi: 10.1038/s41598-022-15526-7 (PMC9287306; doi:10.1038/s41598-022-15526-7)
Supplement: Supplementary file 1 — Supplementary Information 1. [file 41598_2022_15526_MOESM1_ESM.pdf]

Supplemental Figure 1

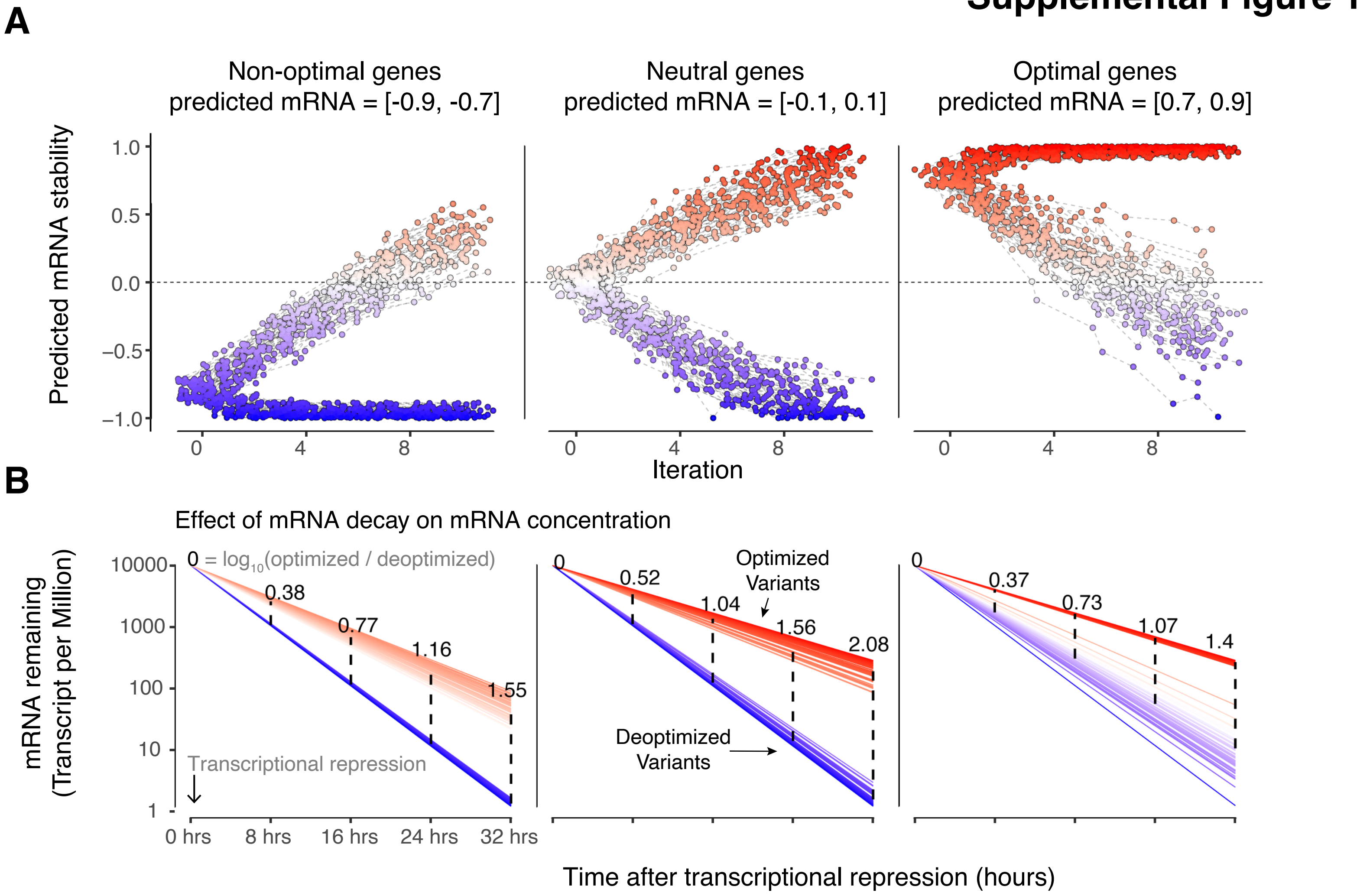

Supplemental Figure 1.

(**A**) Optimization of endogenous genes. Three sets of genes were selected (50 genes in each group): genes that are predicted to be non-optimal (left), genes predicted to be neutral (middle), and genes predicted to be optimal (right). For each of these genes, we ran iCodon to optimize and deoptimize them. The top row shows the iCodon optimization results. The x-axis is the iteration number, and the y-axis is the predicted mRNA stability. The circles connected by a dashed-line show the optimization or deoptimization path for each gene. Gaussian noise was added to the points. (**B**) Simulation of the effect of mRNA stability optimization achieved on mRNA abundance. At time 0 h transcription is inhibited, and the genes decay according to their predicted stability. The lines in red color denote the optimized genes, the lines in blue are the deoptimized genes. mRNA abundance (Transcript per Million) is plotted on a logarithmic scale. The points and dashed lines show the predicted change in expression ($\log_{10}$ fold change) between optimized and deoptimized variants.

Supplemental Figure 2

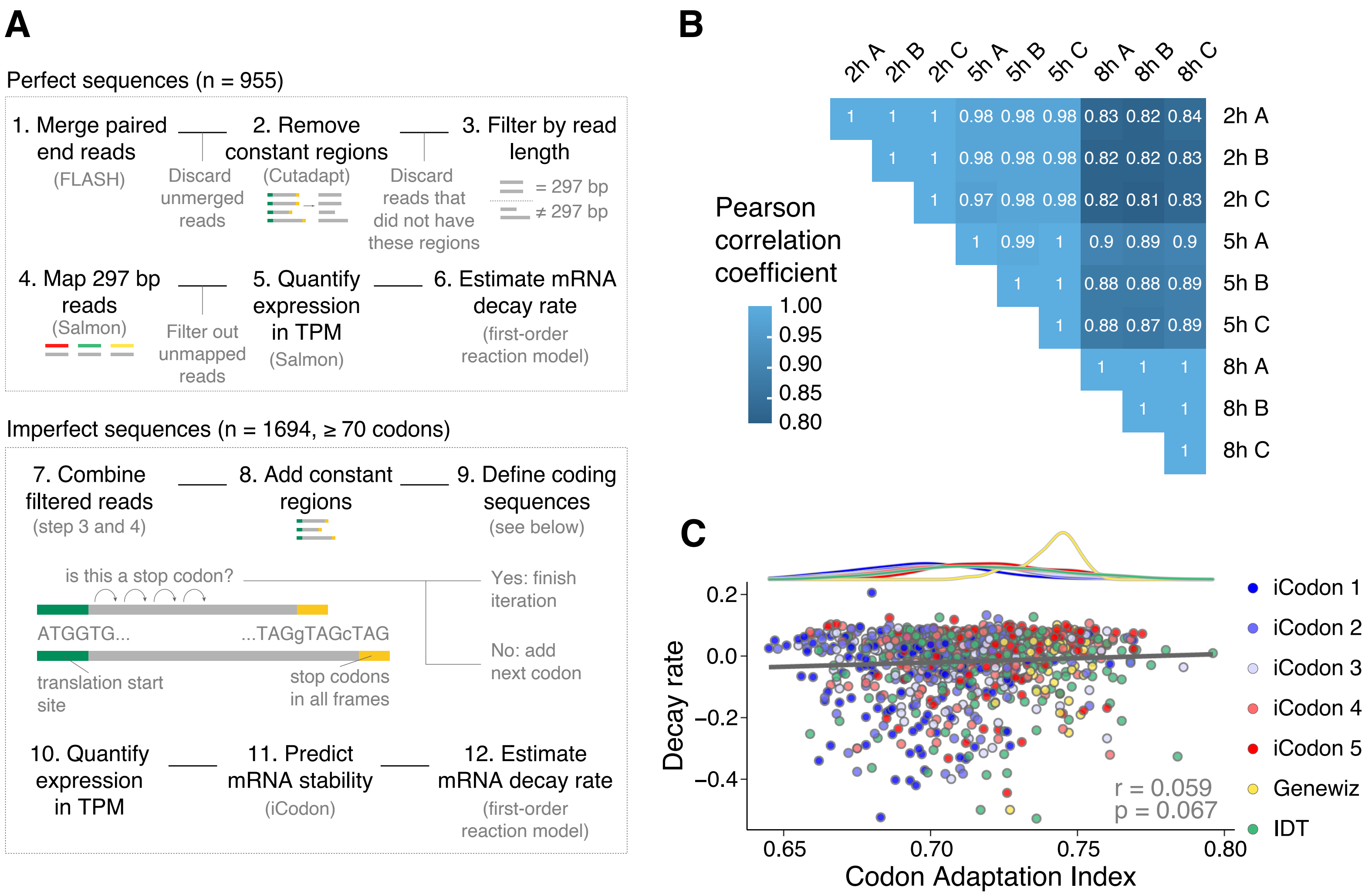

Supplemental Figure 2.

(**A**) Schematic representing the pipeline used to analyze the reporter library. See the methods section. (**B**) Pearson correlation matrix between all 9 library samples. A strong correlation of 1 is observed in almost all replicates between the same time point. All correlations have a p value < 0.05. (**C**) Top: histogram displaying the frequency of Codon Adaptation Index for each group of sequences. Bottom: Scatter plot of Codon Adaptation Index and Decay rate. No correlation is observed between this index and the observed decay.

Supplemental Figure 3

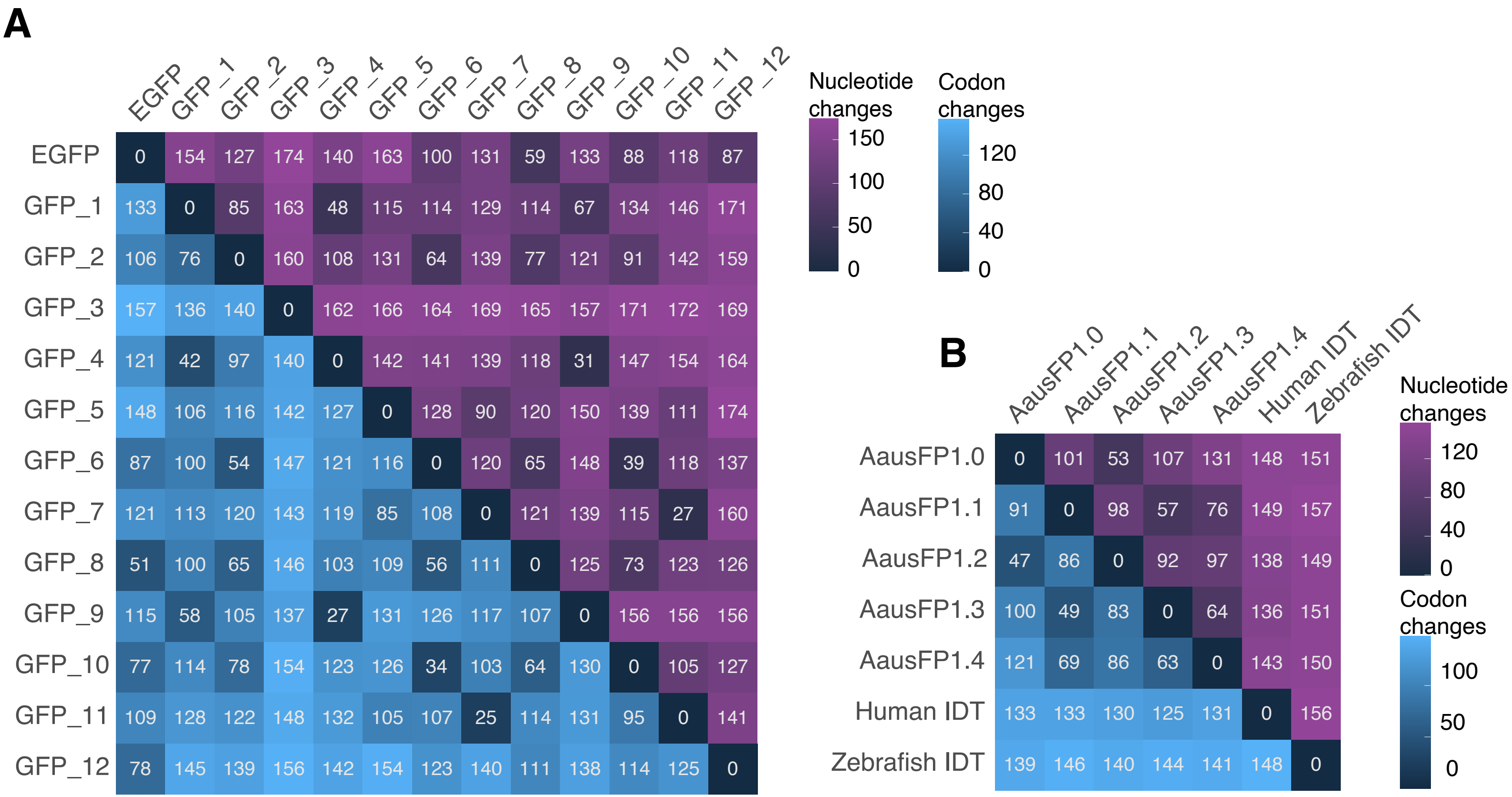

Supplemental Figure 3.

(**A**) Matrix comparing the pairwise distance between the synonymous GFP variants. The lower diagonal entries show the distance in codon changes and the upper diagonal entries in nucleotide changes. (**B**) Same as in panel A for AausFP1variants.
